# Recognition of Drivers’ Hard and Soft Braking Intentions Based on Hybrid Brain-Computer Interfaces

**DOI:** 10.34133/2022/9847652

**Published:** 2022-07-19

**Authors:** Jiawei Ju, Aberham Genetu Feleke, Longxi Luo, Xinan Fan

**Affiliations:** ^1^ School of Mechanical Engineering, Beijing Institute of Technology, Beijing, China; ^2^ Beijing Machine and Equipment Institute China

## Abstract

In this paper, we propose simultaneous and sequential hybrid brain-computer interfaces (hBCIs) that incorporate electroencephalography (EEG) and electromyography (EMG) signals to classify drivers’ hard braking, soft braking, and normal driving intentions to better assist driving for the first time. The simultaneous hBCIs adopt a feature-level fusion strategy (hBCI-FL) and classifier-level fusion strategies (hBCIs-CL). The sequential hBCIs include the hBCI-SE1, where EEG signals are prioritized to detect hard braking, and hBCI-SE2, where EMG signals are prioritized to detect hard braking. Experimental results show that the proposed hBCI-SE1 with spectral features and the one-vs-rest classification strategy performs best with an average system accuracy of 96.37% among hBCIs. This work is valuable for developing human-centric intelligent assistant driving systems to improve driving safety and driving comfort and promote the application of BCIs.

## 1. Introduction

Road traffic accidents (RTA) are one of the most important factors causing casualties and economic losses. Traffic accidents cause nearly 1.35 million deaths and 20-50 million injuries every year [1]. Nearly three percent of gross domestic product is consumed as a result of traffic accidents every year, such as medical expenses for injuries and loss of personnel productivity [2]. In addition, with the fast pace of science, technology, and economic development, vehicles on the road are increasing year by year, and RTA is predicted to be the fifth factor leading to death in 2030 [1, 3].

An intelligent driver assistance system (IDAS) can indirectly influence vehicle control by notifying drivers of possible emergencies or directly controlling vehicles after detecting emergencies, effectively improving drivers’ driving safety.

Some IDASs need to detect drivers’ drowsy state [4, 5] and distraction state [6, 7]. Other IDASs depend on driving behavior detection and prediction of driving intentions [8, 9]. For example, when drivers encounter an emergency during driving, they need to perform emergency (hard) braking. If an IDAS can detect drivers’ hard braking intention in advance, it can directly control vehicles to take hard braking. Braking intention prediction is an important part of IDAS. In this study, braking is a specific behavior that slows or stops the vehicle. The braking can be classified into hard braking and soft braking. Hard braking refers to the behavior in which the driver presses the pedal hard to quickly decrease the vehicle speed in face of an emergency during driving. In contrast, soft braking refers to the behavior in which drivers press the pedal softly to slowly decrease the vehicle speed.

The input information of IDASs mainly consists of vehicle and surrounding-related, behavior-related, and biological signal-related information. The vehicle and surrounding environment information mainly comes from vehicle parameters and traffic information [10–13]. Driver behavior-related information can be obtained mainly by monitoring the activities of drivers’ feet, limbs, and heads [14, 15]. Biological information includes electroencephalography (EEG) signals and electromyography (EMG) signals [16, 17]. Because of the early occurrence of EEG signals, EEG signal-based brain-computer interfaces (BCIs) have been applied to driver behavior research [18–20]. Although these BCIs have made great progress in braking intention detection, the detection performance is not stable because of the properties of EEG signals.

A hybrid brain-computer interface (hBCI) is an effective scheme that can address the shortcomings of EEG-based BCIs, such as low stability, poor performance, and insufficient reliability [21–23]. According to how the signals are combined, the hBCIs fall into two modes. The first mode of hBCIs combines two or more kinds of EEG signals, such as event-related desynchronization (ERD), event-related synchronization (ERS), and P300 [24, 25]. Another mode of hBCIs combines EEG and other signals, such as EMG signals and ECG signals [26–28].

Existing studies of braking intention detection based on hBCIs can be summarized as follows. Haufe et al. [17] studied drivers’ hard braking intention detection in a virtual driving scene by collecting EEG signals, EMG signals, and vehicle information during driving simulation. Then, they detected upcoming hard braking intention by the hBCI model that combines EEG signals, EMG signals, and vehicle information. Their experimental results showed that their hBCI system could recognize hard braking intention 130 ms faster than the model based on the pedal deflection. They set similar car following tasks in the real environment to verify whether there are differences between the virtual environment and the real environment. Then, they collected the same kind of signals and used the same method to recognize hard braking. They confirmed that the detection performance of hard braking in a virtual environment was similar to that in reality [19]. In addition, Kim et al. [29] considered soft braking; they set several stimuli for the same braking intention in a virtual environment. Their model combined EEG signals, EMG signals, and brake pedal information for binary classification of the three driving intentions that are normal, soft, and hard braking in pairs and achieved better performance than BCI. Teng et al. [16] set pedestrians cross the road within a short distance ahead of the vehicle in the virtual driving environment to induce drivers’ hard braking. EEG signals were collected during driving to detect hard braking. They concluded that the detection system was able to recognize hard braking intentions 420 milliseconds after the onset of the emergency situation with a system accuracy of nearly 94%. On this basis, Bi et al. [30] proposed an hBCI model that combined EEG signals and environment information to detect emergency braking intention, which improved the detection performance and reduced the false alarm rate.

However, the above-mentioned detection methods are developed to recognize the hard braking intention from normal driving or soft braking intentions. To promote these detection methods of hard braking intention more applicable in realistic driving situations, our previous study [31] proposed an EEG-based detection method to distinguish hard braking, soft braking, and normal driving intentions. Experimental results suggested the feasibility of this detection method. However, the performance of this detection method was not good. The offline testing average accuracy of the three classes of driving intentions based on spectral features was 70.93%.

To address this problem, in this paper, we aim to develop simultaneous and sequential hBCIs based on EEG and EMG signals to recognize hard braking, soft braking, and normal driving intentions. The contribution of this paper is that it is the first work to use the fusion of EEG and EMG signals to recognize hard braking, soft braking, and normal driving intentions.

The remaining of the article is organized as below: Section 2 introduces the experiment of this study. Section 3 introduces the methodologies. Section 4 presents the results. Section 5 presents the discussion and conclusion of this study.

## 2. Experiment

### 2.1. Subjects

Thirteen subjects aged from 24 to 30 participated in the experiment. The subjects were required to ensure adequate sleep, not drink any substances such as alcohol and coffee that may affect the experiment, and signed the experiment consent before the experiment. The experiment was strictly abided by the 2013 Helsinki Declaration.

### 2.2. Experimental Procedure

**Figure 1:**
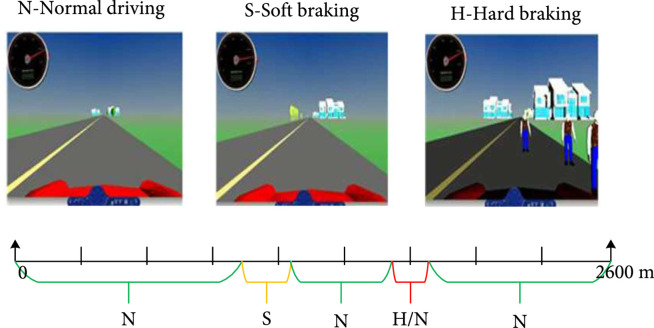
Experimental protocol and virtual scenario.

The experiment was conducted in a virtual environment, as shown in Figure 1. The environment included a 2600 m straight runway, multiple houses, and numerous pedestrians. The driving behaviors involved in this study included hard braking, soft braking, and normal driving. The specific implementation process of the experiment was as follows. At the start of the experiment, subjects maintained normal driving and kept the vehicle driving forward without any unrelated driving behaviors. When the vehicle reached the location 1000 meters away from the starting point, the vehicle speed slowly increased to 120 km/h. As the vehicle speed reached about 120 km/h, subjects entered the soft braking stage and slowed the vehicle to an appropriate speed range between 80 km/h and 100 km/h. After completion of soft braking, subjects resumed normal driving. When the vehicle traveled from 1700 to 2000 meters from the starting point, three pedestrians appeared at random locations on the right side of the path. In addition, when the vehicle reaches a position of 30 meters away from the pedestrian, the pedestrian may begin to cross the road randomly. Subjects then entered the hard braking stage and stepped on the brake pedal firmly to avoid a collision with the crossing pedestrians. After completion of hard braking, subjects resumed the normal driving stage again. If no pedestrian crossed the road, subjects maintained the normal driving to the end.

Each subject conducted 60 trials, and thirty were target trials with hard braking. To avoid the influence of fatigue on our study, we performed the experiments on two different days.

## 3. Methods

### 3.1. System Framework

**Figure 2:**
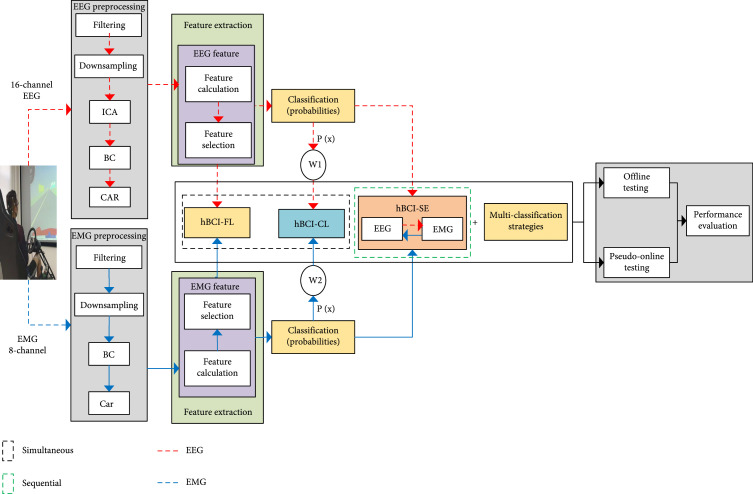
The system framework of the multiclassification detection model based on simultaneous and sequential hBCIs.

Figure 2 shows the framework of the multiclassification detection model based on simultaneous and sequential hBCIs. It includes data acquisition, EEG and EMG signal preprocessing, EEG and EMG signal feature extraction and classification, fusion strategies of simultaneous and sequential hBCIs, multiclassification strategies, offline and pseudoonline testing, and performance assessment.

### 3.2. Data Acquisition

We adopted the 32-channel amplifier of the SYMTOP company to collect sixteen criterion channels EEG signals (P3, P8, P7, P4, Pz, C4, Cz, C3, O1, Oz, O2, F4, Fz, F3, T7, and T8) according to international 10-20 system [32]. Meanwhile, the same acquisition device was used to collect EMG signals to ensure the time synchronization of EEG and EMG signals. The EMG signals were acquired from eight muscles (soleus, external gastrocnemius, tibialis anterior, rectus femoris, biceps brachii, internal gastrocnemius, medial femoral, and lateral femoral) [33]. In addition, we used the other two electrodes to collect the left ear and right ear signals and used the mean value of the left ear and right ear signals as the reference potential. The amplifier had a sampling frequency of 1000 Hz, a six-order Butterworth bandpass filter of 0-120 Hz, and a notch filter of 50 Hz.

### 3.3. EEG and EMG Signal Preprocessing

EEG signal preprocessing: first, the target frequency band (0.5-60 Hz) of EEG signals was obtained by using a sixth-order Butterworth bandpass filter [16]. Second, the signals were downsampled to 200 Hz. Third, we decomposed the EEG data into multiple independent components by independent component analysis (ICA), which is an effective algorithm for removing random blink and EMG artifacts [34]. We applied sixfold cross-validation for training and testing. During the training, it is a manual process to remove artifacts by ICA, and the unmixing matrix is obtained. However, during the testing (i.e., the online detection), the unmixing matrix can be automatically applied [30]. Finally, we eliminated signal drift through the baseline correction (BC) by subtracting the mean value of the first 100 ms data of the corresponding channel to obtain the EEG signals without signal drift. We eliminated the common noise of all channels through the common average reference (CAR) by subtracting the mean value of the corresponding position of all channel data to obtain the EEG signals without common noise.

EMG signal preprocessing: first, the target frequency band (15-90 Hz) was obtained by applying a sixth-order Butterworth bandpass filter [17]. Second, the filtered EMG signals were downsampled to 200 Hz to synchronize with EEG signals. Finally, we eliminated EMG signal drift through BC and reduced the common noise by CAR.

### 3.4. EEG and EMG Feature Extraction and Classification

Feature extraction of EEG and EMG signals:

(i) The common spatial pattern (CSP) was used to project 16-channel EEG data and 8-channel EMG data into m virtual channels of EEG data and M virtual channels of EMG data

The CSP projection process of EEG signals:

(a) We calculated the covariance matrix of each EEG sample, as expressed as follows:

(1)E=XtXtTtraceXtXtT,

where E is the covariance matrix of $X\left(t\right)$, $X\left(t\right)$ is the preprocessed EEG sample, and $X{\left(t\right)}^{T}$ is the transposed matrix of $X\left(t\right)$.

(b) We calculated the average covariance matrices of target and nontarget samples; the mixed covariance matrix, and the expression is as follows:

(2)Em=Et¯+En¯,

where $E_{m}$ represents the mixed covariance matrix of target and nontarget samples and $\bar{E_{t}}$ and $\bar{E_{n}}$ represent the covariance matrix of the target and nontarget samples, respectively.

(c) We decomposed the mixed covariance matrix, which can be described as

(3)Em=FmλmFmT,

where $F_{m}$ represents the eigenvector matrix and $\lambda_{m}$ represents the eigenvalue matrix.

(d) We whiten the mixed covariance matrix, and the expression is as follows:

(4)G=λm−1FmT,(5)Ew=GEmGT,

where $F_{m}$ represents the eigenvector matrix and $\lambda_{m}$ represents the eigenvalue diagonal matrix.

(e) The eigenvalue of $E_{w}$ is equal to 1 with the transformation of G. In addition, according to the expressions (2) and (5), we can obtain

(6)Ht=GEt¯GT,Hn=GEn¯GT,

where $H_{t}$ and $H_{n}$ have the same eigenvector.

(f) We decomposed $H_{t}$ and $H_{n}$ as follows:

(7)Ht=JλtJT,Hn=JλnJT,λt+λn=I,

where J represents the common eigenvector of $H_{t}$ and $H_{n}$. The larger the value of $\lambda_{t}$, and the smaller the value of $\lambda_{n}$. Therefore, this property is conducive to distinguishing the two types of samples.

(g) We calculated the projection matrix of CSP, which can be expressed as follows:

(8)We=JTGT,

(h) We can obtain the EEG sample after projection. Moreover. We can obtain the EMG sample after projection in the same way, which can be described as follows:

(9)Xet=WeXt,Xet=WeXt,

7where $W_{e}$ is the projection matrix for EEG signals, $X_{e}\left(t\right)=\left[{xe}_{1}\left(t\right),{xe}_{2}\left(t\right),\cdots ,{xe}_{m}\left(t\right)\right]$, and ${xe}_{m}\left(t\right)$ is the mth channel of projected EEG data. The projection matrix for EMG signals is $W_{m}$, and $Y\left(t\right)$ is the preprocessed EMG signals. $Y_{m}\left(t\right)=\left[{ym}_{1}\left(t\right),{ym}_{2}\left(t\right),\cdots ,{ym}_{M}\left(t\right)\right]$, where ${ym}_{M}\left(t\right)$ is the Mth virtual channel of EMG data.

(ii) We calculated the temporal and spectral features of EEG and EMG signals

EEG signal feature calculation: hard braking, soft braking, and normal driving are specific in EEG amplitude and power spectral density function [31]. Therefore, the projected EEG signal amplitude was used as the original EEG temporal feature $X_{e}\left(t\right)$. The original spectral feature $X_{e}\left(f\right)$ is the power spectral density function obtained from $X_{e}\left(t\right)$ by fast Fourier transform (FFT).

EMG signal feature calculation: hard braking, soft braking, and normal driving are specific in EMG envelope amplitude and power spectral density function [33]. Therefore, the original EMG temporal feature $Y_{lm}\left(t\right)$ was obtained from $Y_{m}\left(t\right)$ by applying the linear envelope (rectification followed by a second-order Butterworth low-pass filter with a cut-off frequency of 2), and $Y_{m}\left(f\right)$ is the original spectral feature obtained from $Y_{m}\left(t\right)$ through FFT.

(iii) We selected EEG signal features and EMG signal features by using the distance correlation coefficient (DCC) to reduce the calculation time of the model [35].

For the spectral feature selection of EEG signals, there are four steps as follows:

(a) We calculated the norm distance of each feature pair:

(10)aqij=Aqi−Aqj, i,j=1,2,⋯n,bqij=Bqi−Bqj, i,j=1,2,⋯n,

where the normal distance of the ith feature of $A^{q}$ and the jth feature of $A^{q}$ is ${a^{q}}_{ij}$, $A^{q}=\left[t_{q}\left(1\right),\cdots t_{q}\left(k\right),n_{q}\left(1\right),\cdots n_{q}\left(r\right)\right]$, and $t_{q}\left(k\right)$ and $n_{q}\left(r\right)$ represent the qth characteristic of $X_{e}\left(f\right)$ in the kth target sample and the rth nontarget sample, respectively. The normal distance of the ith feature of $B^{q}$ and the jth feature of $B^{q}$ is ${b^{q}}_{ij}$, $B^{q}=\left[1_{q}\left(1\right),\cdots 1_{q}\left(k\right),-1_{q}\left(1\right),\cdots -1_{q}\left(r\right)\right]$, $-1_{q}\left(r\right)$ is the category label of $n_{q}\left(r\right)$, and $1_{q}\left(k\right)$ is the category label of $t_{q}\left(k\right)$.

(b) We centralized the normal distance matrices of the qth feature matrix and the reference matrix, respectively

(11)Cqij=aqij−aqi¯−aqj¯+aq¯,Dqij=bqij−bqi¯−bqj¯+bq¯,

where ${C^{q}}_{ij}$ is the centralized normal distance between the ith and the jth feature of $A^{q}$, $\bar{{a^{q}}_{i}}$ is the average value of ith row of its norm distance matrix, $\bar{{a^{q}}_{j}}$ is the average value of jth column of its norm distance matrix, and $\bar{a^{q}}$ is the average value of its norm distance matrix. ${D^{q}}_{ij}$ is the centralized normal distance between ith and jth feature of $B^{q}$, $\bar{{b^{q}}_{i}}$ is the average value of ith row of its norm distance matrix, $\bar{{b^{q}}_{j}}$ is the average value of jth column of its norm distance matrix, and $\bar{b^{q}}$ is the average value of its norm distance matrix.

(c) The square covariance of $C^{q}$ and $D^{q}$ was calculated in Equation (12), and the variance of $C^{q}$ and the variance of $D^{q}$ were calculated in Equations (13) and (14), respectively. Then, the distance correlation coefficient between $A^{q}$ and $B^{q}$ was obtained in (15).

(12)dCovn2Aq,Bq=1n2∑i=1n∑j=1nCqijDqij,

(13)dVarn2Aq=dCovn2Aq,Aq=1n2∑i,j=1nCqij2,

(14)dVarn2Bq=dCovn2Bq,Bq=1n2∑i,j=1nDqij2,

(15)dCorAq,Bq=dCovAq,BqdVarAqdVarBq.

(d) We selected W features with high distance correlation coefficients from $X_{e}\left(f\right)$. Similarly, we selected W features from $X_{e}\left(t\right)$, $Y_{lm}\left(t\right)$, and $Y_{m}\left(f\right)$, respectively

EEG and EMG signal classification: in related studies [16, 17, 30], regularized linear discriminant analysis (RLDA) has achieved a good decoding performance. Therefore, we used it as the classifier. The classifier can be expressed as follows.

(16)y=ψTx,

where y is the output value, x represents the selected temporal or spectral features, $\psi =\sum_{\omega }^{\prime }/\left(v_{1}-v_{2}\right)$ is the matrix of projection, and $v_{1}$ and $v_{2}$ are the average values of features of the two classes samples, respectively. $\sum_{\omega }^{\prime }$ is computed by

(17)∑ω′=1−λ∑ω+λωI,

where the regularized parameter λ takes the value in the range from 0.1 to 1 with a step size of 0.1, $\sum_{\omega }$ is the within-class matrix, $\omega =\text{trace}\left(\sum_{\omega }\right)/g$, g is the dimension of $\sum_{\omega }$, and I represents the identity matrix.

Furthermore, the classification probability of each sample was obtained

(18)P=12+12∗minymax,y−threymax−thre, y≥thre,12−12∗thre‐maxymin,ythre−ymin, y<thre,

where P is the classification probability, thre is the threshold value, and ${\text{y}}_{\max}$ and $y_{\min}$ are the maximum and minimum values of y associated with training samples.

### 3.5. Fusion Strategies of Simultaneous and Sequential hBCI

The target of fusion strategies is to take full advantage of EEG and EMG signals. In this research, the simultaneous hBCI models used EEG and EMG signals together in every part of the models. Meanwhile, the sequential hBCI models used EEG signals in one part of the models, and EMG signals in the other part.

In Figure 2, simultaneous hBCIs included hBCI-FL and hBCI-CL, whereas the sequential hBCI included hBCI-SE. In this study, we applied the static fusion rules for all models.

For the hBCI-FL with the feature-level fusion strategy, the selected EEG and EMG signal features were normalized by the min-max normalization. Then, the two signal features were concatenated and fed to the classifier to obtain the classification result.

For the hBCI-CL with the classifier-level fusion strategy, we calculated the classification probabilities of the EEG-based classifier and EMG-based classifier according to (18). The two classification probabilities were combined in different ways to obtain the final classification result. In this study, three classifier fusion strategies were adopted as follows.

The hBCI-CL1 adopted the “AND” fusion strategy. If both classification results based on the EEG classifier and EMG classifier are both targets, the output result is the target. Otherwise, the output result is nontarget.

The hBCI-CL2 used the “OR” fusion strategy. If one of the output results of the EEG-based classifier and EMG-based classifier is the target, the final output result is the target. Otherwise, the output result is nontarget.

The hBCI-CL3 adopted the Naïve Bayesian fusion strategy [36]. It used the conditional probability $P\left(G\left|F_{1},F_{2}\right.\right)$ by (19) to combine the decisions from EEG-based and EMG-based classifiers.

(19)PGF1,F2∝PGPF1,F2G,

where G denotes the class (target or nontarget), $F_{1}$ and $F_{2}$ are the output results of the EEG-based classifier and EMG-based classifier, respectively.

The Bayesian fusion strategy assumed that EEG and EMG are independent in the detection process before the results output. Then, we coupled EEG and EMG at the decision level. Although the EEG and EMG signals are tightly coupled together, we can separately use them to build models. Therefore, we assumed that the two signal sources used in the detection process before the final result output are independent in our models [36]. We can obtain

(20)PF1,F2G=PF1GPF2G,

According to (19) and (20), we can have

(21)Gout=argmaxPG=hPF1G=hPF2G=h,

with $h\in \left(\text{target},\text{nontarget}\right)$. $P\left(F_{1}\left|G\right.=h\right)$ and $P\left(F_{2}\left|G\right.=h\right)$ are the classification probabilities obtained by the EEG-based classifier and EMG-based classifier, respectively, and $G_{\text{out}}$ is the output result. Assume that the prior probabilities of the two classes $P\left(G=h\right)$ are the same.

The hBCI-SE1 model used EEG signals to recognize the hard braking intention from soft braking and normal driving intentions and used EMG signals to implement other binary classifiers. In contrast, the hBCI-SE2 model used EMG signals to recognize hard braking and EEG signals to implement other binary classifiers.

### 3.6. Multiclassification Strategies

In this study, the one-vs-rest and the decision tree strategies were adopted for multiclass classification. The one-vs-rest classification strategy decomposed the three-class classification into three parallel binary classifications, including normal driving versus the rest, soft braking versus the rest, and hard braking versus the rest. For the one-vs-rest classification strategy, the final result $R_{a}$ is obtained according to the maximum value of all binary classifiers. The decision rule expression is as follows:

(22)Ra=argi=1,2,3maxyi.

The decision tree strategy decomposed the three-class classification into a cascade of two binary classifications. The first binary classifier was first used to classify hard braking from soft braking and normal driving. If the classification was not hard braking, the second one was used to classify soft braking and normal driving. For the decision rules, we compared $y_{1}$ with $K_{1}$ (threshold of the first classifier) and $y_{2}$ with $K_{2}$ to obtain the final result $R_{b}$. If $y_{1}>K_{1}$, $R_{b}$ is hard braking. Otherwise, it is nonhard braking. Then, if $y_{2}>K_{2}$, $R_{b}$ is soft braking. Otherwise, it is normal driving.

### 3.7. Offline and Pseudoonline Testing and Performance Assessment

For the offline three-class classification, first, we extracted one-second data (16-channel EEG data and 8-channel EMG data) before the onset of the pedal deflection of hard and soft braking from each trial as one hard and soft braking samples, respectively. In this way, we obtained 30 hard-braking samples and soft-braking samples for each subject. Second, one-second data were extracted randomly from the interval of 11 to 4 seconds before the pedal deflection of hard braking from each trial as one normal driving sample. Likewise, we obtained 30 normal driving samples for each subject. The detection model was evaluated by the sixfold cross-validation.

**Figure 3:**
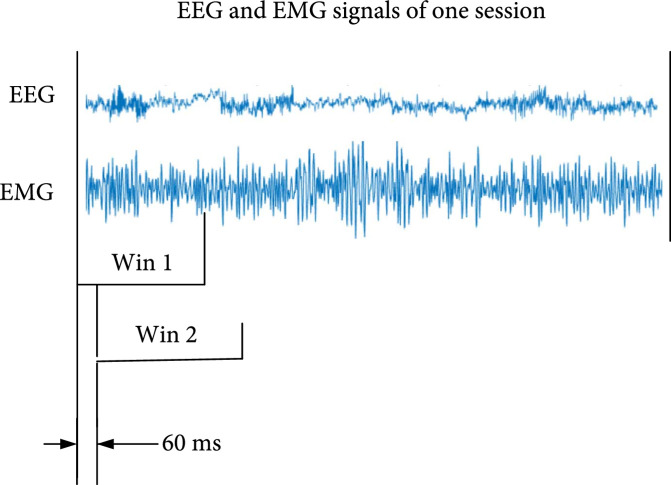
Pseudoonline process illustration.

Pseudoonline testing is similar to online testing. However, the testing data need to be collected in advance. The pseudoonline testing process is shown in Figure 3. First, we set the value of W, m, and M for each subject according to the W, m, and M values obtained in the offline analysis. Second, we fed EEG and EMG signals of one-second window size (win 1) to the detection model to obtain the detection result. Third, we move the window by a fixed step size of 60 ms to get a new window (win 2) and fed the EEG and EMG signals corresponding to win 2 to the detection model to obtain another detection result. Fourth, repeat the third operation until the end of one session. Finally, we obtained the test results of all experimental trials for each subject.

For offline analysis, we used the three classes of driving samples extracted from the test trials for the detection model testing. In comparison, for the pseudoonline analysis, the one-second test samples extracted from the test trials were continuously input to the detection model with a fixed step size.

Since the pseudoonline testing is different from the offline testing, we adopted separate evaluation indexes. The accuracy was used as the performance index for the offline multiclassification, whereas the system accuracy was used as the performance index. The system accuracy is described as

(23)Sa=1−Fal+Hit_e+Hit_s3,

where the false positive rate is Fal and Hit_e and Hit_s are the hit rate of hard and soft braking, respectively. Note that the soft and hard braking intentions are considered to be hit if the soft and hard braking intentions are detected within 1.2 s after the soft braking and hard braking stimulation appearance, respectively [16, 17, 30].

Furthermore, we used the advanced time to verify the feasibility of our detection model, which represents the time interval between the onset of the pedal deflection and the time instant when the detection system issues the braking intention.

## 4. Results

In this section, first, we decoded three driving intentions by using all simultaneous hBCI models to obtain the optimal simultaneous hBCI model. Second, we decoded the three driving intentions using different sequential hBCI models to obtain the optimal sequential hBCI model. Third, the proposed models were evaluated by comparing the optimal simultaneous hBCI and the optimal sequential hBCI with models based on single EEG signals or EMG signals. Fourth, the optimal sequential hBCI, the optimal sequential hBCI, and models based on single EEG signals or EMG signals were used for pseudoonline testing. Finally, to further validate the proposed methods, we compared them with those related methods in other studies.

### 4.1. Offline Multiclassification Performance Based on Simultaneous hBCIs

(i) One-vs-rest vs. decision tree in temporal features

**Figure 4:**
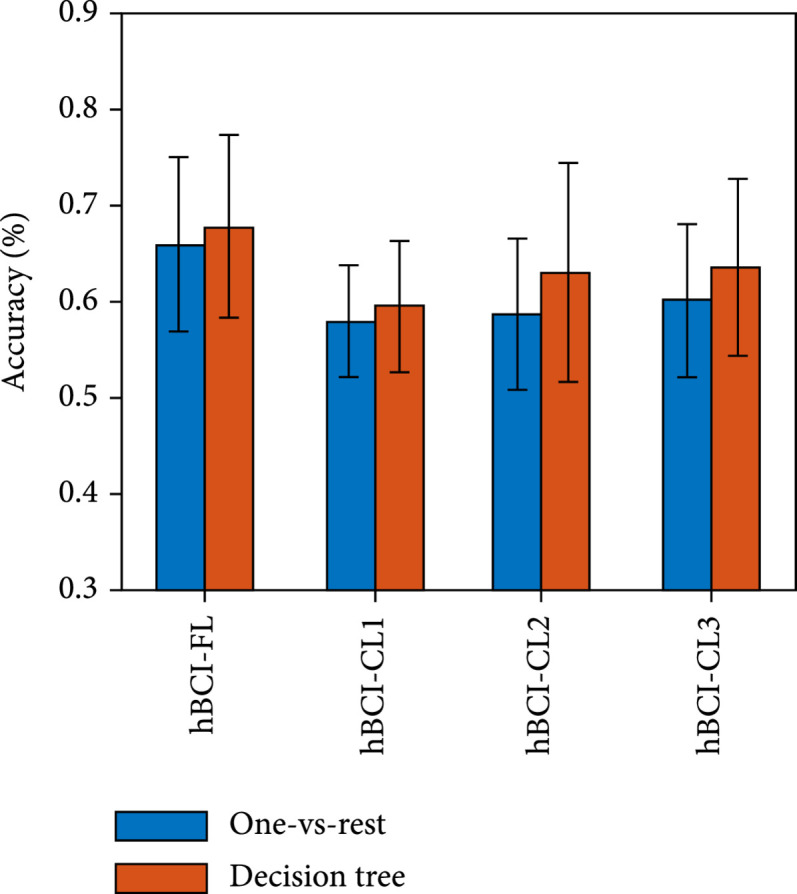
Mean±standard deviation (SD) of the accuracy of four simultaneous hBCI models based on temporal features in decoding the three driving intentions. The hBCI-FL represents hBCI models with fusion strategy at the feature level. The hBCI-CL represents models with fusion strategy at the classifier level, including hBCI-CL1, hBCI-CL2, and hBCI-CL3 based on different classifier fusion strategies.

Figure 4 presents the accuracy of the simultaneous hBCI models based on temporal features in decoding hard braking, soft braking, and normal driving. The result shows that the decision tree achieves better decoding performance than the one-vs-rest.

(ii) One-vs-rest vs. decision tree in spectral features

**Figure 5:**
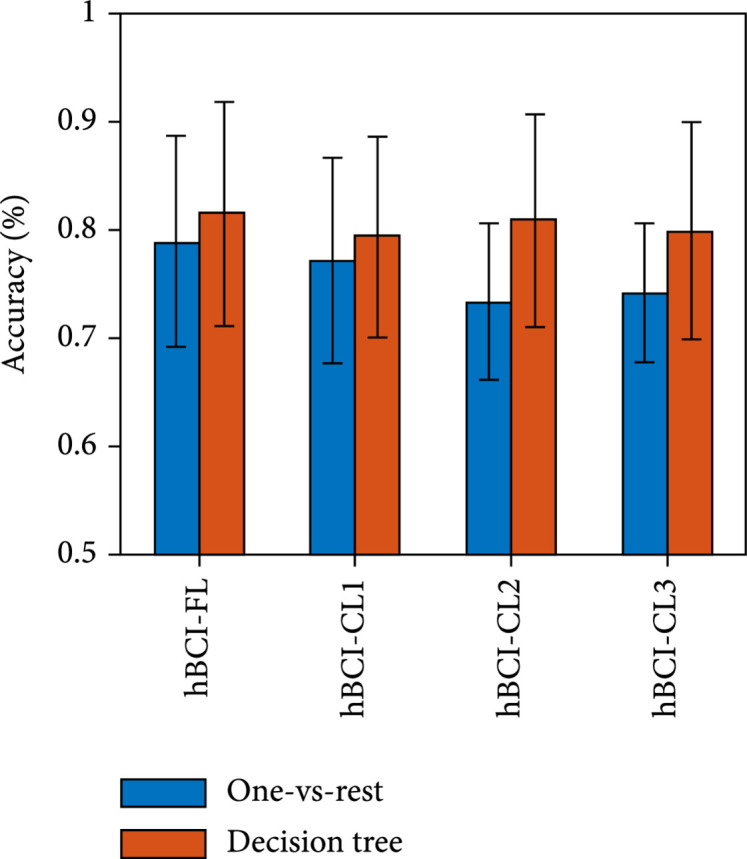
Mean±SD of the accuracy of four simultaneous hBCI models based on spectral features in decoding the three driving intentions. The hBCI-FL represents hBCI models with fusion strategy at the feature level. The hBCI-CL represents models with fusion strategy at the classifier level, including hBCI-CL1, hBCI-CL2, and hBCI-CL3 based on different classifier fusion strategies.

Figure 5 presents the accuracy of the simultaneous hBCI models based on spectral features in decoding hard braking, soft braking, and normal driving. The result shows that the decision tree achieves better decoding performance than the one-vs-rest.

(iii) Temporal features vs. spectral features

**Figure 6:**
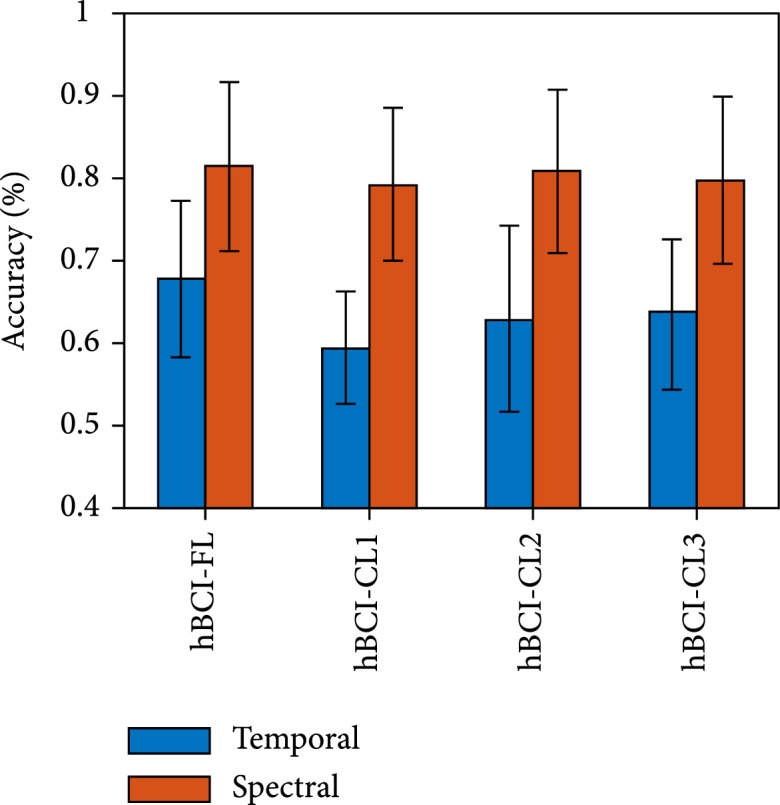
Mean±SD of the accuracy of four simultaneous hBCI models in decoding the three driving intentions. The hBCI-FL represents hBCI models with fusion strategy at the feature level. The hBCI-CL represents models with fusion strategy at the classifier level, including hBCI-CL1, hBCI-CL2, and hBCI-CL3 based on different classifier fusion strategies.

Figure 6 shows the performance of simultaneous hBCI models based on the decision tree classification strategy in decoding hard braking, soft braking, and normal driving. The result shows that the spectral feature-based hBCI models obtained better performance than temporal feature-based hBCI models.

In addition, the decoding performance of the hBCI-FL adopting spectral features and the decision tree classification strategy produced the best result in terms of the average performance. Therefore, it was selected as the optimal simultaneous hBCI model.

### 4.2. Offline Multiclassification Performance Based on Sequential hBCIs

**Figure 7:**
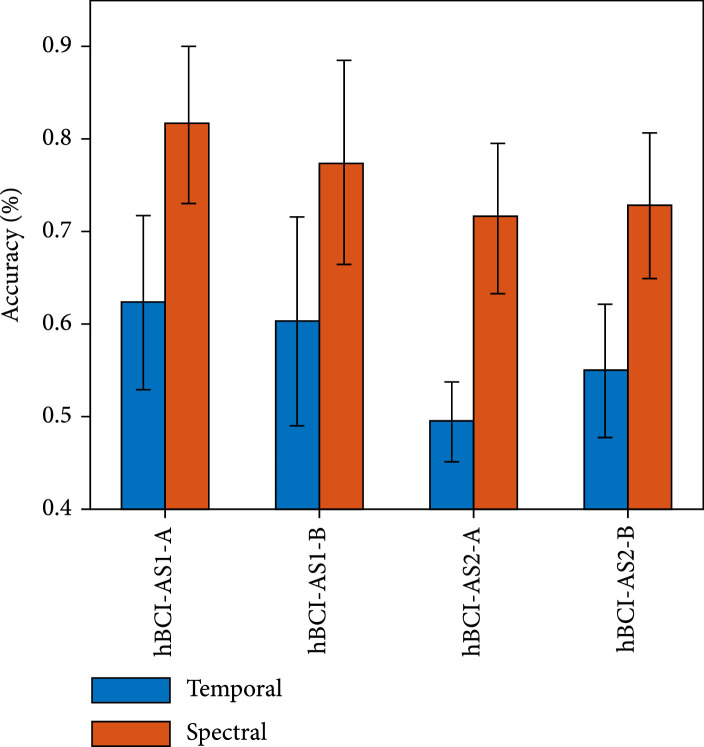
Mean±SD of the accuracy of four sequential hBCI models in decoding the three driving intentions. The hBCI-SE1 gives priority to EEG signals, and hBCI-SE2 gives priority to EMG signals. The postfix “A” represents the one-vs-rest classification strategy, and the postfix “B” represents the decision tree classification strategy.

Figure 7 presents the performance of sequential hBCI models in decoding hard braking, soft braking, and normal driving. The sequential hBCIs include hBCI-SE1 and hBCI-SE2.

The points that can be extracted from the results:

(i) The sequential hBCIs with spectral features produced results better than those with temporal features
(ii) The decoding performance of hBCI-SE1 was better than that of hBCI-SE2 with the same features
(iii) The hBCI-SE1 model adopting spectral features and the one-vs-rest classification strategy achieved the best decoding performance and thus was chosen as the optimal sequential hBCI model

### 4.3. Offline Multiclassification Performance Comparison between hBCIs Models and Single EEG or EMG Models

(i) Comparison in EEG bands

In the spectral features, the frequency bands of EEG signals included the δ band (0–4 Hz), Θ band (4–8 Hz), α band (8–13 Hz), β band (13–30 Hz), γ band (30–50 Hz), and 0.5-60 Hz [31].

**Figure 8:**
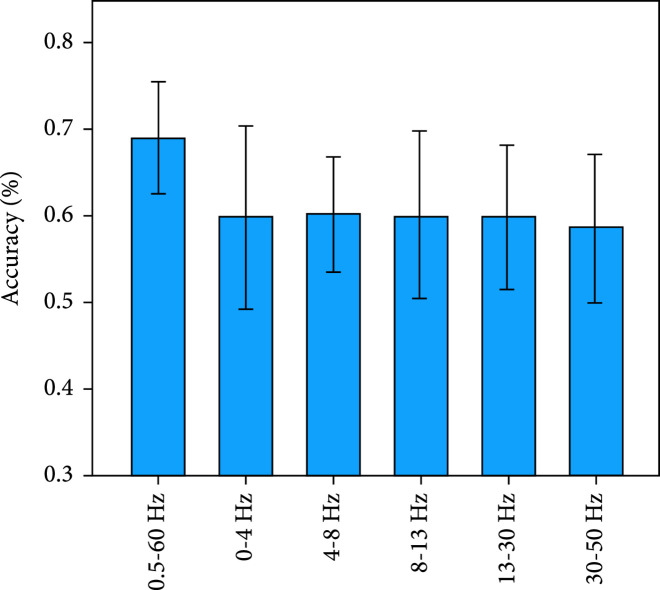
Mean±SD of the accuracy of EEG models based on different spectral features and the one-vs-rest classification strategy in decoding the three driving intentions.

**Figure 9:**
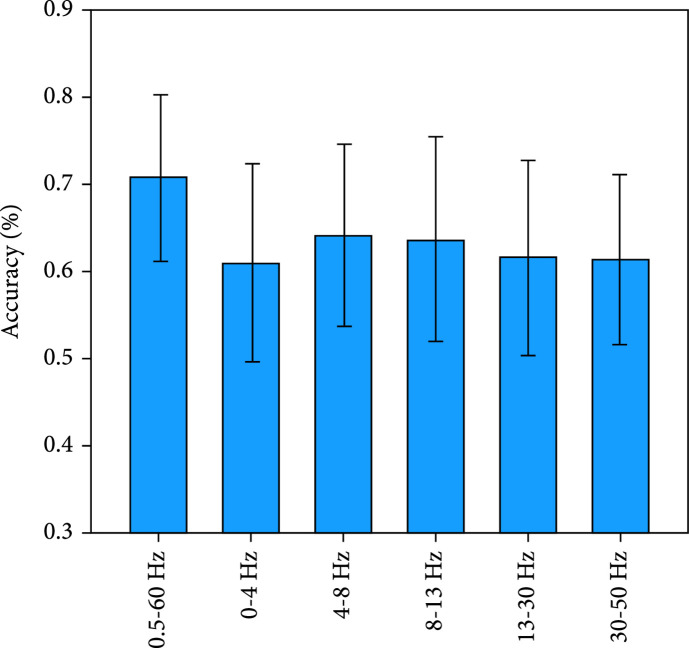
Mean±SD of the accuracy of EEG models based on different spectral features and the decision tree classification strategy in decoding the three driving intentions.

Figure 8 presents the classification accuracy of EEG models based on different spectral features and the one-vs-rest classification strategy in decoding hard braking, soft braking, and normal driving. Besides, Figure 9 presents the classification accuracy of EEG models based on different spectral features and the decision tree classification strategy in decoding the three driving intentions. The result shows that 0.5-60 Hz achieves the best detection performance in the EEG-based models.

(ii) hBCIs models vs. EEG or EMG models

**Figure 10:**
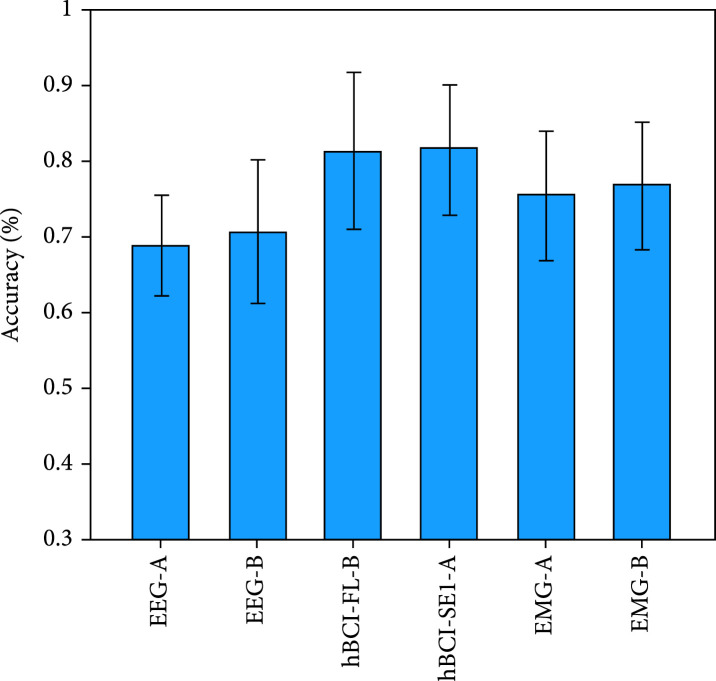
Mean±SD of the accuracy of the optimal simultaneous and sequential hBCI models and single EEG or EMG models in decoding the three driving intentions. The hBCI-FL is the optimal simultaneous hBCI model, and the hBCI-SE1 is the optimal sequential hBCI model. The postfix “A” represents the one-vs-rest classification strategy, and the postfix “B” represents the decision tree classification strategy.

Figure 10 shows the performance of the optimal simultaneous hBCI model, the optimal sequential hBCI model, and models based on single EEG or EMG signals in decoding the three driving intentions.

(a) The single EEG models and single EMG models based on the decision tree classification strategy achieved slightly better decoding performance than those based on the one-vs-rest classification strategy
(b) The decoding performance of the optimal simultaneous model hBCI-FL based on spectral features and decision tree classification strategy (81.45%±10.29%) was similar to that of the optimal sequential model hBCI-SE1 based on spectral features and the one-vs-rest classification strategy (81.54%±8.5%). Furthermore, both optimal simultaneous and sequential hBCI models performed better than the optimal single EEG model based on spectral features and decision tree classification strategy (70.68%±9.52%) and the optimal single EMG model based on spectral features and decision tree classification strategy (76.87%±8.29%).

In addition, Table 1 shows the parameters for virtual channel selection and feature selection in the optimal simultaneous and sequential hBCI models and those in the optimal single EEG and single EMG models for each subject.

**Table 1:** The parameter setting of projected channels and spectral features of different decoding models for decoding the three driving intentions (Note that m (m was chosen from 2 to 16 with an interval of 2) is the number of the virtual channels of EEG signals, M (M was chosen from 2 to 8 with an interval of 2) is the number of virtual channels of EMG signals, W (W was chosen from 10 to 100 with an interval of 10) is the number of selected features of EEG and EMG signals, and the parameters were determined by offline performance. The hBCI-FL represents models that adopt the fusion strategy at the feature level, and the hBCI-SE1 is the sequential model with priority on EEG signals. The postfix “A” represents the one-vs-rest classification strategy, and the postfix “B” represents the decision tree classification strategy).

Subject no.	hBCI-FL-B			hBCI-SE1-A			EEG-B		EMG-B	
	Virtual channel (m)	Virtual channel (M)	Feature number (W)	Virtual channel (m)	Virtual channel (M)	Feature number (W)	Virtual channel (m)	Feature number (W)	Virtual channel (M)	Feature number (W)
S1	16	2	10	2	6	50	8	100	6	70
S2	14	2	10	6	2	70	8	20	4	20
S3	2	8	90	6	2	80	4	10	4	10
S4	8	6	30	6	6	70	10	100	6	70
S5	16	6	10	2	8	20	8	90	2	90
S6	2	2	40	2	8	50	14	60	2	30
S7	14	8	30	6	6	20	6	10	8	30
S8	2	6	100	10	8	90	16	10	8	100
S9	8	4	70	10	4	100	10	70	6	20
S10	6	2	10	6	4	70	4	20	2	10
S11	2	4	30	6	4	90	12	20	2	40
S12	14	8	90	10	4	20	10	20	4	10
S13	6	2	10	16	2	50	16	70	2	90

### 4.4. Pseudoonline Multiclassification Performance Based on Simultaneous and Sequential hBCIs

**Figure 11:**
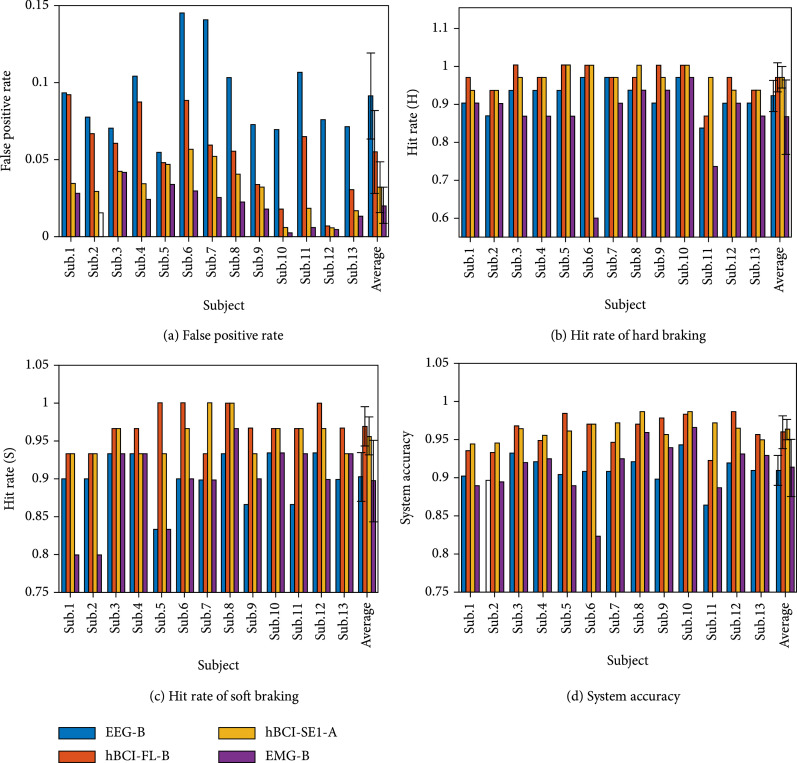
The system accuracy in detecting hard and soft braking by using the four optimal models. The EEG-B is the EEG-based detection model with the decision tree classification strategy, the hBCI-FL-B is the simultaneous model at the feature level fusion strategy with the decision tree classification strategy, the hBCI-SE1-A is the sequential model that gives priority to EEG signals with the one-vs-rest classification strategy, and the EMG-B is the EMG-based detection model with the decision tree classification strategy (note that all the above detection models are based on spectral features).

Figure 11 shows the system accuracy of the three driving intentions detection by using the optimal simultaneous hBCI model (hBCI-FL-B), the optimal sequential hBCI model (hBCI-SE1-A), the optimal single EEG model (EEG-B), and the optimal single EMG model (EMG-B). The EEG-B model had a system accuracy of 90.98%±1.92%, and the EMG-B model had a system accuracy of 91.37%±3.72%. The hBCI-FL-B model achieved 4.67% higher system accuracy ($F\left(1,12\right)=20.70$, p=0.001) than that of the EMG-B model. The system accuracy of hBCI-SE1-A model was 5% higher ($F\left(1,12\right)=25.80$, p<0.001) than that of the EMG-B model. We adopted the one-way repeated-measures analysis of variance (ANOVA) to evaluate the difference level and considered the model as a factor, and the reference value of the significant difference was 0.05.

In addition, the advanced time of hard and soft braking detection by using the hBCI-FL-B model were 268.83 ± 85.19 ms and 256.45 ± 64.43 ms, respectively. The advanced time of hard and soft braking detection by using the hBCI-SE1-A model was 300.14 ± 82.50 ms and 193.54 ± 45.68 ms, respectively. All pseudoonline detection performance is shown in Table 2.

**Table 2:** Pseudoonline detecting performance. Note that the results were averaged across all subjects. The hBCI-FL-B represents models that adopt the fusion strategy at the feature level and the decision tree classification strategy, the hBCI-SE1-A represents models that adopt the sequential fusion strategy with priority on EEG signals and the one-vs-rest classification strategy, the EEG-B is the EEG-based detection model using the decision tree classification strategy, and the EMG-B is the EMG-based detection model using the decision tree classification strategy. SA represents the system accuracy, AT (H) represents the advanced time of hard braking relative to behavior, and AT (S) represents the advanced time of soft braking relative to behavior.

	hBCI-FL-B	hBCI-SE1-A	EEG-B	EMG-B
SA (%)	96.04±2.12	96.37±1.37	90.98±1.92	91.37±3.72
AT (H) (ms)	268.83±85.19	300.14±82.50	328.64±80.48	185.69±53.87
AT (S) (ms)	256.45±64.43	193.54±45.68	288.22±74	157.74±45.20

### 4.5. Binary Classification Performance Based on hBCI

For the offline binary classification, we adopted the hBCI-FL to build binary decoding models. We extracted the three classes of driving samples according to the sample selection method of the offline three-class classification. Each decoding model was evaluated by using sixfold cross-validation.

Temporal and spectral features were used to build the hBCI-FL binary decoding models. Table 3 presents the binary classification results. There were three binary classification tasks (hard braking versus soft braking, hard braking versus normal driving, and soft braking versus normal driving). From Table 3, we can see that the binary classification performance of models based on spectral features was 6% to 15% higher than models based on temporal features. The binary classification performance index used in this paper was the area under the curve (AUC); AUC was used to compare the performance with the related study.

**Table 3:** Decoding performance of binary classification based on temporal and spectral features. Note that the results were averaged across all subjects. H is hard braking, S is soft braking, and N is normal driving. The italicized data represent temporal features, and the data in bold represent spectral features.

	H	S	N
H		*80.33%*	*86.10%*
S	**96.26%**		*90.74%*
N	**95.23%**	**94.10%**	

## 5. Discussion and Conclusion

In this paper, we compared and analyzed several simultaneous and sequential hBCI models, which used spectral or temporal features and the one-vs-rest or decision tree classification strategy for multiclassification of the three driving intentions. The offline results showed that hBCI-FL adopting spectral features and the decision tree classification strategy achieved the best decoding performance among the simultaneous hBCIs. And the hBCI-SE1 based on spectral features and the one-vs-rest classification strategy achieved the best decoding performance among the sequential hBCIs. In addition, we compared the optimal simultaneous hBCI model and the optimal sequential hBCI model with models based on single EEG or EMG signals. The results demonstrated that the optimal simultaneous and sequential hBCIs both performed significantly better than those based on single EEG or EMG signals. Furthermore, pseudoonline testing was conducted and produced results in good agreement with the offline testing results.

This research promotes the development of intelligent assistant systems. On the one hand, the upcoming braking intentions can be predicted using our method. On the other hand, this study explored the simultaneous hBCIs and sequential hBCIs, respectively. It can benefit not only the study of drivers’ behavior but also the study of drivers’ states.

Compared with the related study [29], the proposed hBCI methods achieved better performance in binary classification. In decoding hard braking from normal driving, our method performed better with an AUC of 95.23%, compared to the method based on EEG signals in [29] with an AUC of about 90%. The AUC of our methods in decoding soft braking versus normal driving reached 94.10%. In comparison, the AUC of the method based on EEG signals was about 69% [29]. Similarly, our methods showed better results (AUC of 96.26%) in decoding soft and hard braking than the methods based on EMG signals (AUC of about 74%) [29]. Moreover, existing studies did not perform multiclassification, whereas our system accuracy in recognizing hard barking, soft braking, and normal driving intentions reached 96.37%. However, it should be noted that the comparative studies and this research have differences in the simulated environment, subjects, and collecting device. Thus, the direct performance comparison may be unfair.

There are still some limitations in this study. First, stimuli to induce hard braking and soft braking are not diverse. Hard braking and soft braking were induced by the pedestrian crossing the road at short distances and overspeed, respectively. There are a variety of stimuli for inducing hard braking and soft braking. For example, the sudden braking of the leading vehicle at a close distance can induce hard braking. Sudden vehicle stop at a long distance can induce soft braking. More stimuli for hard and soft braking should be included in the experiment. Moreover, the stimuli to induce the mixed situation of hard braking and soft braking should also be considered. Second, we did not consider the impact of the subjects’ differences in our research, such as gender, height, driving experience, and sitting posture. These differences and their impacts on detecting hard and soft braking can be studied to make our method more applicable. Third, the data collection method used in our study requires subjects to wear the acquisition device, which is inconvenient for subjects. Wireless acquisition equipment developed recently may solve this issue. Moreover, fewer channels are more appropriate from the perspective of usability but may compromise the accuracy.

In the future, we will address the limitations mentioned above. We will consider various stimuli, explore the impact of the subjects’ differences on recognizing braking intentions, improve the existing data acquisition method, and explore more effective features and fusion strategies to improve the performance. Overall, existing and future research can help improve driving safety and comfort in this field.

## Data Availability

The data used to support the findings of this study are available from the corresponding author upon request.

## References

[1] World Health Organization *Road traffic injuries*, World Health Organization, 2018, http://www.who.int/mediacentre/factsheets/fs358/en/.

[2] A. M. Almeshal, M. R. Alenezi, and A. K. Alshatti, “Accuracy assessment of small unmanned aerial vehicle for traffic accident photogrammetry in the extreme operating conditions of Kuwait,” *Information*, vol. 11, no. 9, p. 442, 2020

[3] T. Y. Chen, and R. C. Jou, “Using HLM to investigate the relationship between traffic accident risk of private vehicles and public transportation,” *Transportation Research Part A Policy and Practice*, vol. 119, pp. 148–161, 2019

[4] M. Srilatha, “Accident avoidance and detection system using LabVIEW,” *International Journal of Advanced Trends in Computer Science and Engineering*, vol. 9, no. 4, pp. 5314–5319, 2020

[5] A. Tiwari, and P. Kumar, “Driver drowsiness detection for accident prevention using EEG signal,” *Middle-East Journal of Scientific Research*, vol. 24, no. 7, pp. 2338–2341, 2016

[6] D. Xu, H. Zhao, F. Guillemard, S. Geronimi, and F. Aioun, “Aware of scene vehicles—probabilistic modeling of car-following behaviors in real-world traffic,” *IEEE Transactions on Intelligent Transportation Systems*, vol. 20, no. 6, pp. 2136–2148, 2019

[7] N. Li, and C. Busso, “Predicting perceived visual and cognitive distractions of drivers with multimodal features,” *IEEE Transactions on Intelligent Transportation Systems*, vol. 16, no. 1, pp. 51–65, 2015

[8] S. Schnelle, J. Wang, H. Su, and R. Jagacinski, “A personalizable driver steering model capable of predicting driver behaviors in vehicle collision avoidance maneuvers,” *IEEE Transactions on Human-Machine Systems*, vol. 47, no. 5, pp. 625–635, 2017

[9] W. Wang, J. Xi, and D. Zhao, “Learning and inferring a driver's braking action in car-following scenarios,” *IEEE Transactions on Vehicular Technology*, vol. 67, no. 5, pp. 3887–3899, 2018

[10] A. Kashevnik, I. Lashkov, and A. Gurtov, “Methodology and mobile application for driver behavior analysis and accident prevention,” *IEEE Transactions on Intelligent Transportation Systems*, vol. 21, no. 6, pp. 2427–2436, 2020

[11] D. Wang, X. Pei, L. Li, and D. Yao, “Risky driver recognition based on vehicle speed time series,” *IEEE Transactions on Human-Machine Systems*, vol. 48, no. 1, pp. 63–71, 2018

[12] S. G. Christopoulos, S. Kanarachos, and A. Chroneos, “Learning driver braking behavior using smartphones, neural networks and the sliding correlation coefficient: road anomaly case study,” *IEEE Transactions on Intelligent Transportation Systems*, vol. 20, no. 1, pp. 65–74, 2019

[13] W. Wang, J. Xi, A. Chong, and L. Li, “Driving style classification using a semisupervised support vector machine,” *IEEE Transactions on Human-Machine Systems*, vol. 47, no. 5, pp. 650–660, 2017

[14] S. Y. Cheng, S. Park, and M. M. Trivedi, “Multi-spectral and multi-perspective video arrays for driver body tracking and activity analysis,” *Computer Vision and Image Understanding*, vol. 106, no. 2-3, pp. 245–257, 2007

[15] A. Rangesh, and M. M. Trivedi, “HandyNet: a one-stop solution to detect, segment, localize & analyze driver hands,” in *IEEE/CVF Conference on Computer Vision and Pattern Recognition Workshops (CVPRW)*, USA, 2018, pp. 18–22

[16] T. Teng, L. Bi, and Y. Liu, “EEG-based detection of driver emergency braking intention for brain-controlled vehicles,” *IEEE Transactions on Intelligent Transportation Systems*, vol. 19, no. 6, pp. 1766–1773, 2018

[17] S. Haufe, M. Treder, M. Gugler, M. Sagebaum, G. Curio, and B. Blankertz, “EEG potentials predict upcoming emergency brakings during simulated driving,” *Journal of Neural Engineering*, vol. 8, no. 5, article 056001, 2011 10.1088/1741-2560/8/5/056001 21799241

[18] I. H. Kim, J. W. Kim, S. Haufe, and S. W. Lee, “Detection of multi-class emergency situations during simulated driving from ERP,” in *International Winter Workshop on Brain-Computer Interface (BCI)*, Gangwon, Korea (South), 2013, pp. 18–20

[19] S. Haufe, J. W. Kim, I. H. Kim, A. Sonnleitner, M. Schrauf, G. Curio, and B. Blankertz, “Electrophysiology-based detection of emergency braking intention in real-world driving,” *Journal of Neural Engineering*, vol. 11, no. 5, article 056011, 2014 10.1088/1741-2560/11/5/056011 25111850

[20] Z. Khaliliardali, R. Chavarriaga, L. A. Gheorghe, and J. . R. Millán, “Action prediction based on anticipatory brain potentials during simulated driving,” *Journal of Neural Engineering*, vol. 12, no. 6, article 066006, 2015 10.1088/1741-2560/12/6/066006 26401885

[21] M. Xu, J. Han, Y. Wang, T. P. Jung, and D. Ming, “Implementing over 100 command codes for a high-speed hybrid brain-computer interface using concurrent P300 and SSVEP features,” *IEEE Transactions on Biomedical Engineering*, vol. 67, no. 11, pp. 3073–3082, 2020 32149621 10.1109/TBME.2020.2975614

[22] Y. Yu, Z. Zhou, Y. Liu, J. Jiang, E. Yin, N. Zhang, Z. Wang, Y. Liu, X. Wu, and D. Hu, “Self-paced operation of a wheelchair based on a hybrid brain-computer interface combining motor imagery and P300 potential,” *IEEE Transactions on Neural Systems and Rehabilitation Engineering*, vol. 25, no. 12, pp. 2516–2526, 2017 29220327 10.1109/TNSRE.2017.2766365

[23] M. Kim, B. H. Kim, and S. Jo, “Quantitative evaluation of a low-cost noninvasive hybrid interface based on EEG and eye movement,” *IEEE Transactions on Neural Systems and Rehabilitation Engineering*, vol. 23, no. 2, pp. 159–168, 2015 25376041 10.1109/TNSRE.2014.2365834

[24] L. W. Ko, O. Komarov, and S. C. Lin, “Enhancing the hybrid BCI performance with the common frequency pattern in dual-channel EEG,” *IEEE Transactions on Neural Systems and Rehabilitation Engineering*, vol. 27, no. 7, pp. 1360–1369, 2019 31180893 10.1109/TNSRE.2019.2920748

[25] A. Katyal, and R. Singla, “A novel hybrid paradigm based on steady state visually evoked potential & P300 to enhance information transfer rate,” *Biomedical Signal Processing and Control*, vol. 59, article 101884, 2020

[26] S. He, Y. Zhou, T. Yu, R. Zhang, Q. Huang, L. Chuai, M. U. Mustafa, Z. Gu, Z. L. Yu, H. Tan, and Y. Li, “EEG- and EOG-based asynchronous hybrid BCI: a system integrating a speller, a web browser, an E-mail client, and a file explorer,” *IEEE Transactions on Neural Systems and Rehabilitation Engineering*, vol. 28, no. 2, pp. 519–530, 2020 31870987 10.1109/TNSRE.2019.2961309

[27] X. Chai, Z. Zhang, K. Guan, Y. Lu, G. Liu, T. Zhang, and H. Niu, “A hybrid BCI-controlled smart home system combining SSVEP and EMG for individuals with paralysis,” *Biomedical Signal Processing and Control*, vol. 56, no. 2, article 101687, 2020

[28] Y. Zhou, S. He, Q. Huang, and Y. Li, “A hybrid asynchronous brain-computer interface combining SSVEP and EOG signals,” *IEEE Transactions on Biomedical Engineering*, vol. 67, no. 10, pp. 2881–2892, 2020 32070938 10.1109/TBME.2020.2972747

[29] I. H. Kim, J. W. Kim, S. Haufe, and S. W. Lee, “Detection of braking intention in diverse situations during simulated driving based on EEG feature combination,” *Journal of neural engineering*, vol. 12, no. 1, article 016001, 2015 10.1088/1741-2560/12/1/016001 25426805

[30] L. Bi, H. Wang, T. Teng, and C. Guan, “A novel method of emergency situation detection for a brain-controlled vehicle by combining EEG signals with surrounding information,” *IEEE Transactions on Neural Systems & Rehabilitation Engineering*, vol. 26, no. 10, pp. 1926–1934, 2018 30188835 10.1109/TNSRE.2018.2868486

[31] J. Ju, L. Bi, and A. G. Feleke, “Noninvasive neural signal-based detection of soft and emergency braking intentions of drivers,” *Biomedical Signal Processing and Control*, vol. 72, article 103330, 2022

[32] H. Steinmetz, G. Fürst, and B. U. Meyer, “Craniocerebral topography within the international 10-20 system,” *Electroencephalography and Clinical Neurophysiology*, vol. 72, no. 6, pp. 499–506, 1989 2471619 10.1016/0013-4694(89)90227-7

[33] J. Ju, and L. Bi, “Driving Intention Decoding from EMG Signals for Human-Vehicle Interaction,” in *IEEE International Conference on Real-time Computing and Robotics (RCAR)*, Japan, 2020, pp. 28–29

[34] Teng *Neural Signature and Decoding of Driver’s Emergency Braking Intention and Application of Decoding Model*, Dept. Beijing University of Technology, China, 2018, Ph. D. dissertation.

[35] J. Kundrata, D. Fujimoto, Y. Hayashi, and A. Barić, “Comparison of Pearson correlation coefficient and distance correlation in correlation power analysis on digital multiplier,” in *2020 43rd International Convention on Information, Communication and Electronic Technology (MIPRO)*, Croatia, 2020, pp. 146–151

[36] R. Leeb, H. Sagha, R. Chavarriaga, and J. . R. Millán, “A hybrid brain–computer interface based on the fusion of electroencephalographic and electromyographic activities,” *Journal of Neural Engineering*, vol. 8, no. 2, article 025011, 2011 10.1088/1741-2560/8/2/025011 21436524

